# Local‐to‐Nonlocal Second‐Harmonic Generation from Electrically Tunable Intersubband Polaritonic Metasurfaces

**DOI:** 10.1002/advs.202518776

**Published:** 2025-11-29

**Authors:** Jaesung Kim, Hyeongju Chung, Seongjin Lee, Gerhard Boehm, Mikhail A. Belkin, Jongwon Lee

**Affiliations:** ^1^ Department of Electrical Engineering Ulsan National Institute of Science and Technology (UNIST) Ulsan 44919 Republic of Korea; ^2^ Walter Schottky Institute Technical University of Munich Am Coulombwall 4 85748 Garching Germany

**Keywords:** angle‐multiplexed optics, dual tunability, electrical tuning, local‐to‐nonlocal process, nonlinear metasurfaces, nonlocal resonance, second‐harmonic generation

## Abstract

Nonlinear optical metasurfaces enable subwavelength control of light‐matter interactions, yet simultaneous tunability of harmonic signal intensity and spectral response remains a fundamental challenge. Here, a local‐to‐nonlocal second harmonic (SH) generation process is presented, enabled by an electrically tunable polaritonic metasurface, allowing independent control of the SH spectral peak wavelength and intensity. The metasurface combines a localized surface plasmon resonance at the fundamental frequency with a transverse magnetic guided‐mode resonance at the SH frequency. By engineering modal overlap within a multiple quantum well layer, voltage‐controlled modulation of SH intensity and angle‐controlled spectral tuning is achieved, demonstrating two decoupled degrees of freedom associated with local and nonlocal modes. Angle‐resolved nonlinear reflection measurements confirm the independent tunability of the metasurface, validating the separation of excitation and emission pathways. This hybrid approach provides a general framework for nonlinear metasurfaces with enhanced flexibility and functional control, paving the way for applications in nonlinear signal processing, angle‐multiplexed photonics, and entangled photon‐pair generation for quantum optics.

## Graphical abstract

A local‐to‐nonlocal SHG process is demonstrated on an electrically tunable metasurface, combining an LSPR at the FF with a TM‐GMR at the SH to achieve independent dual tunability. Through modal‐overlap engineering of the proposed scheme, a high‐χ^(2)^ MQW meta‐atom delivers strong nonlinearity while achieving 2‐DoF SH control with minimal trade‐offs, advancing the functionality of nonlinear nonlocal metasurfaces.

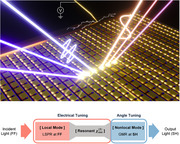

## 1 Introduction

Nonlinear optical metasurfaces have attracted considerable attention owing to their relaxed phase‐matching requirements compared to bulk nonlinear crystals, as well as their ability to engineer nonlinear optical responses at the subwavelength scale.^[^ ^1^, ^2^, ^3^ ^]^ By leveraging strong near‐field enhancement in nanocavities, these metasurfaces can exhibit significantly enhanced second‐ or third‐order nonlinear susceptibilities (χ^(2)^ or χ^(3)^),^[^ ^4^, ^5^ ^]^ enabling a wide range of applications including nonlinear beam shaping,^[^ ^6^, ^7^, ^8^ ^]^ circular dichroism,^[^ ^9^, ^10^, ^11^ ^]^ holography,^[^ ^12^, ^13^, ^14^ ^]^ optical imaging,^[^ ^15^, ^16^, ^17^ ^]^ and nonclassical light generation.^[^ ^18^, ^19^, ^20^ ^]^ Recent advances have further shown that the nonlinear response of metasurfaces can be actively modulated through electrical^[^ ^21^, ^22^ ^]^ or optical^[^ ^23^, ^24^ ^]^ tuning mechanisms, expanding their potential for reconfigurable and tunable photonic devices. In particular, nonlinear intersubband polaritonic metasurfaces have demonstrated remarkable frequency conversion efficiencies exceeding 0.2% for second harmonic generation (SHG),^[^ ^25^ ^]^ as well as ultrafast electrical modulation of second harmonic (SH) intensity in the mid‐infrared regime.^[^ ^26^ ^]^ These results are achieved by coupling plasmonic resonators–engineered to support doubly resonant local modes– with the resonant intersubband nonlinear optical response of multiple quantum wells (MQWs). While spectral tuning of the intersubband nonlinear response can be achieved through the quantum‐confined Stark effect (QCSE),^[^ ^27^ ^]^ the overall spectral tunability of the metasurface's effective nonlinear response remains fundamentally limited by the fixed local resonant modes of structurally rigid resonators that cannot be dynamically reconfigured.

Meanwhile, increasing attention has been directed toward nonlocal metasurfaces that leverage collective resonances arising from interactions between spatially distributed meta‐atoms. These nonlocal responses provide high quality (Q) factors, strong near‐field enhancement, and broad spectral tunability as a function of in‐plane momentum,^[^ ^28^, ^29^ ^]^ rendering them promising platforms for nonlinear optical applications.^[^ ^30^ ^]^ Indeed, a range of demonstrations–including up‐conversion imaging,^[^ ^31^ ^]^ broadband SHG,^[^ ^32^ ^]^ terahertz (THz) sources,^[^ ^33^ ^]^ and spatially entangled photon‐pair generation^[^ ^34^ ^]^–have been reported. However, most implementations to date rely on materials with either intrinsically weak nonlinear susceptibilities (as in dielectric structures) or low nonlinear current generation (as in plasmonic structures), due to the stringent requirement for low optical loss in sustaining nonlocal resonances. As a result, these systems typically exhibit extremely low power conversion efficiencies, often limited to the photon‐counting level, and necessitate ultrahigh peak pump intensities from femtosecond lasers. Nonlinear nonlocal metasurfaces for SHG remain largely unexplored, particularly for utilizing their angle‐dependent resonances to tune the SH spectrum. A distinctive contribution here is demonstrating that, unlike prior work focused on high‐Q resonances, nonlocal metasurfaces can enable angle‐dependent SHG spectral control. Conventional designs, however, tie both FF and SH modes to the same tuning parameter–the incident angle–thus limiting independent control over spectral tuning and nonlinear modulation.

**Figure 1:**
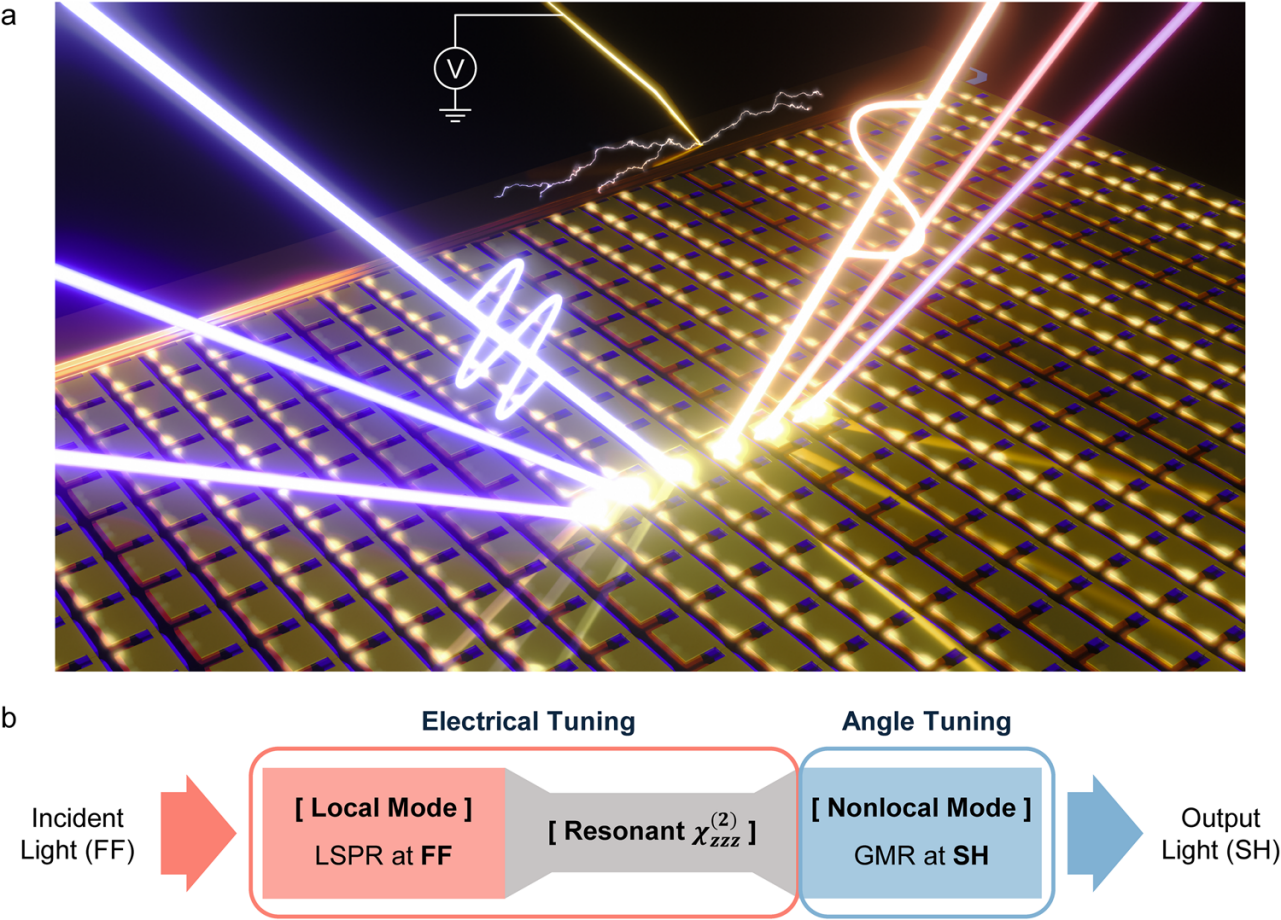
Concept of the local‐to‐nonlocal SHG process. a) Schematic illustration of dual‐tunability from a nonlinear intersubband polaritonic metasurface for SHG. b) Diagram of the local‐to‐nonlocal SHG process. The incident FF light is absorbed via a localized mode, while the generated SH light is out‐coupled through a nonlocal mode. In the designed metasurface, the local and nonlocal modes are independently controlled by electrical bias and incident angle, respectively.

In this work, we propose for the first time a hybrid SHG scheme that combines a local resonant mode at the fundamental frequency (FF) with a nonlocal resonant mode at the SH frequency, enabling a novel local‐to‐nonlocal frequency conversion process. This approach allows for efficient frequency conversion while simultaneously achieving broadband SHG spectral tunability through variation of the incident angle, as illustrated in **Figure** **1**a. In this configuration, the incident FF light is coupled into a local electromagnetic mode–specifically, a localized surface plasmon resonance (LSPR)–while the SH polarization generated within the MQW layer, which exhibits a strong resonant $\chi_{zzz}^{\left(2\right)}$, is subsequently out‐coupled through a nonlocal mode, namely a guided mode resonance (GMR), as illustrated in Figure 1b. At the FF, electrical tuning of the local mode can also be achieved via QCSE of the intersubband transition (IST). At the SH frequency, angular tuning of the nonlocal GMR enables output SHG spectral control. This local‐to‐nonlocal SHG scheme thus provides two independent tuning knobs–electrical tuning and angle tuning–allowing for independent control over spectral tunability and intensity modulation.

## 2 Results

### 2.1 Metasurface Design

**Figure 2:**
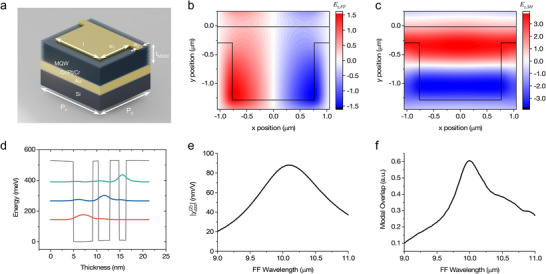
Metasurface design and mode profiles. a) Unit cell structure of the designed metasurface supporting both local and nonlocal modes. Geometric parameters are: P_x_ = 2.1 µm, P_y_ = 1.7 µm, t_MQW_ = 500 nm, l = 1.7 µm, w_1_ = 0.98 µm, and w_2_ = 0.28 µm. b,c) Simulated *E_z_* field distributions of b) the local mode under x‐polarized FF excitation and c) the nonlocal modes under y‐polarized SH excitation, both within the MQW layer. d) Conduction band diagram of the designed MQW structure,^[^ ^39^ ^]^ showing the wavefunctions of three confined electron subbands (E_1_: red, E_2_: blue, and E_3_: green). e) Calculated second order nonlinear susceptibility element $|\chi_{zzz}^{\left(2\right)}|$ of the MQW as a function of the FF wavelength. f) Calculated spectrum of the modal overlap factor as a function of the FF wavelength.

To implement the proposed local‐to‐nonlocal SHG scheme, the meta‐atom unit must simultaneously support a local resonant mode at the FF and a nonlocal resonant mode at the SH frequency. As a starting design point, a 2D rectangular‐patch array with independently tuned length and width can be configured to support a nonlocal mode and a local mode at target wavelengths under different polarizations. Furthermore, to prevent far‐field cancellation of the SH output, careful consideration of the modal overlap between the two resonances is essential.^[^ ^35^ ^]^ To meet these design criteria, we developed a meta‐atom consisting of rectangular gold nanoantenna pads, each connected to a thin metal line on one edge, periodically arranged in a 2D array, as shown in **Figure** **2**a. This geometry supports an LSPR under x‐polarized excitation at the FF, and a transverse magnetic (TM) GMR under y‐polarized excitation at the SH frequency. The resonance wavelength of the LSPR is primarily dictated by the antenna length, *l*, whereas the TM GMR is governed by the MQW thickness, *t*_MQW_ and the period along the y‐direction, *P*_y_. Achieving strong modal overlap is inherently challenging when relying solely on nonlocal modes, due to their lack of spatial field confinement. In contrast, the local‐to‐nonlocal approach introduces an additional degree of freedom in meta‐atom design by enabling intentional field asymmetry through localized resonances. This asymmetry is critical for realizing a non‐zero modal overlap factor, thereby facilitating efficient nonlinear frequency conversion. In MQWs, the second‐order nonlinear susceptibility arising from resonant ISTs is dominated by the $\chi_{zzz}^{\left(2\right)}$ tensor element, which is driven by electric fields oriented along the z‐direction (surface‐normal direction).^[^ ^36^ ^]^ To maximize frequency conversion efficiency, it is therefore essential to generate strong z‐oriented electric fields within the MQW layer and ensure substantial modal overlap of these fields at both the fundamental and second harmonic frequencies.^[^ ^35^ ^]^ Figure 2b,c illustrate the simulated z‐directional electric field distributions within the MQW layer for the two resonant modes. The inclusion of the connecting metal line localizes the FF mode field toward the lower edge of the rectangular antenna pad, producing an asymmetric field profile along the y‐axis, as shown in Figure 2b. In addition to shaping the local field distribution, the metal line also functions as an electrical contact, enabling the application of a vertical bias across the MQW layer and thereby allowing electrical modulation of the nonlinear optical response. At the SH wavelength, the designed metasurface exhibits a first‐order diffraction angle of $\theta =\sin^{-1}\left(\frac{m\lambda_{\mathrm{SH}}}{nP_{y}}\right)=63.1^{\circ }$. The simulated field profile of the GMR shows nodal points at both the center and edges of the meta‐atom, as shown in Figure 2c, indicating that the SH mode corresponds to a ±1st‐order TM GMR.^[^ ^37^, ^38^ ^]^ The guiding reflection angle can be estimated as $\theta =\tan^{-1}\left(\frac{P_{y}/2}{t_{\mathrm{MQW}}}\right)=59.5^{\circ }$, which closely matches the diffraction angle, further confirming the GMR nature of the SH resonance. Unlike in conventional dielectric GMR metasurfaces, the metal‐insulator‐metal architecture used here introduces a π phase shift upon each guided reflection. To satisfy the phase‐matching condition for constructive interference and collective resonance, the GMR mode must exhibit two nodes along the guiding direction–requiring the effective guiding length of the meta‐atom to be doubled in that direction. Moreover, employing a TM‐polarized GMR is crucial for coupling to the z‐polarized SH field component, which is responsible for the $\chi_{zzz}^{\left(2\right)}$ nonlinearity in the MQW structure.

A coupled triple quantum well unit structure was designed using an In_0.53_Ga_0.47_As/Al_0.48_In_0.52_As heterostructure, as illustrated in Figure 2d. The layer sequence of the MQW structure is **5**/4.2/**1.2**/2.5/**2.5**/1.5/**5** nm, where the bolded layers represent Al_0.48_In_0.52_As barriers. The first 4.2 nm well is *n*‐doped to a carrier concentration of 3.5 × 10^18^ cm^−3^. In this work, a 500 nm‐thick MQW layer–comprising 23 repetitions of this unit structure–was employed. This MQW structure exhibits a resonant second‐order nonlinear susceptibility $\chi_{zzz}^{\left(2\right)}$ along the growth (z) direction, with a peak resonant value approaching 90 nm V^−1^ near a wavelength of 10 µm, as shown in Figure 2e. Furthermore, the ISTs between the three spatially separated electron subbands can be actively tuned via the QCSE under an externally applied bias, enabling dynamic electrical control of the SHG intensity. Further details on the characteristics of this MQW structure have been reported in our previous work.^[^ ^39^ ^]^

The effective second‐order susceptibility, $\chi_{yxx}^{(2)\mathrm{eff}}$, of the designed nonlinear polaritonic metasurface can be expressed as:^[^ ^35^ ^]^

(1) $$\begin{array}{ccc} & & \chi_{\mathrm{yxx}}^{\left(2\right)\mathrm{eff}}\left(V,k_{y}\right)\\ & & \;\approx \chi_{\mathrm{zzz}}^{\left(2\right)\mathrm{MQW}}\left(V\right)\cdot \left[\int_{v_{\mathrm{MQW}}}\frac{E_{z}^{2\omega }\left(2k_{y}\right)}{E_{y,\mathrm{inc}}^{2\omega }}\frac{E_{z}^{\omega }\left(V\right)}{E_{x,\mathrm{inc}}^{\omega }}\frac{E_{z}^{\omega }\left(V\right)}{E_{x,\mathrm{inc}}^{\omega }}\mathrm{dv}\right]/v_{\mathrm{unit}} \end{array}$$

where the volume integral in the square brackets in Equation (1) is referred to as the modal overlap integral. The term $E_{z}^{\omega \;or\;2\omega }\left(V\;or\;2k_{y}\right)/E_{i,inc}^{\omega \;or\;2\omega }$ represents the enhancement factor of the *E*_*z*_ field in the MQW region as a function of the y‐directional in‐plane momentum *k_y_* and applied voltage *V*, normalized to the incident electric field, $E_{i,inc}^{\omega \;or\;2\omega }$, polarized along the *i*‐direction (x or y) at the FF ω or the SH frequency 2ω, respectively. *v*_unit_ is the unit cell volume, and *v*_MQW_ is the MQW layer volume within the metasurface unit cell. Owing to the non‐centrosymmetric field product distribution between the local and nonlocal modes (Figure 2b,c), the designed meta‐atom yields a non‐zero modal overlap factor under the normal pump incidence (i.e. *k_y_* =  0), as shown in Figure 2f.

### 2.2 Linear Characterization of Dual Tunability

**Figure 3:**
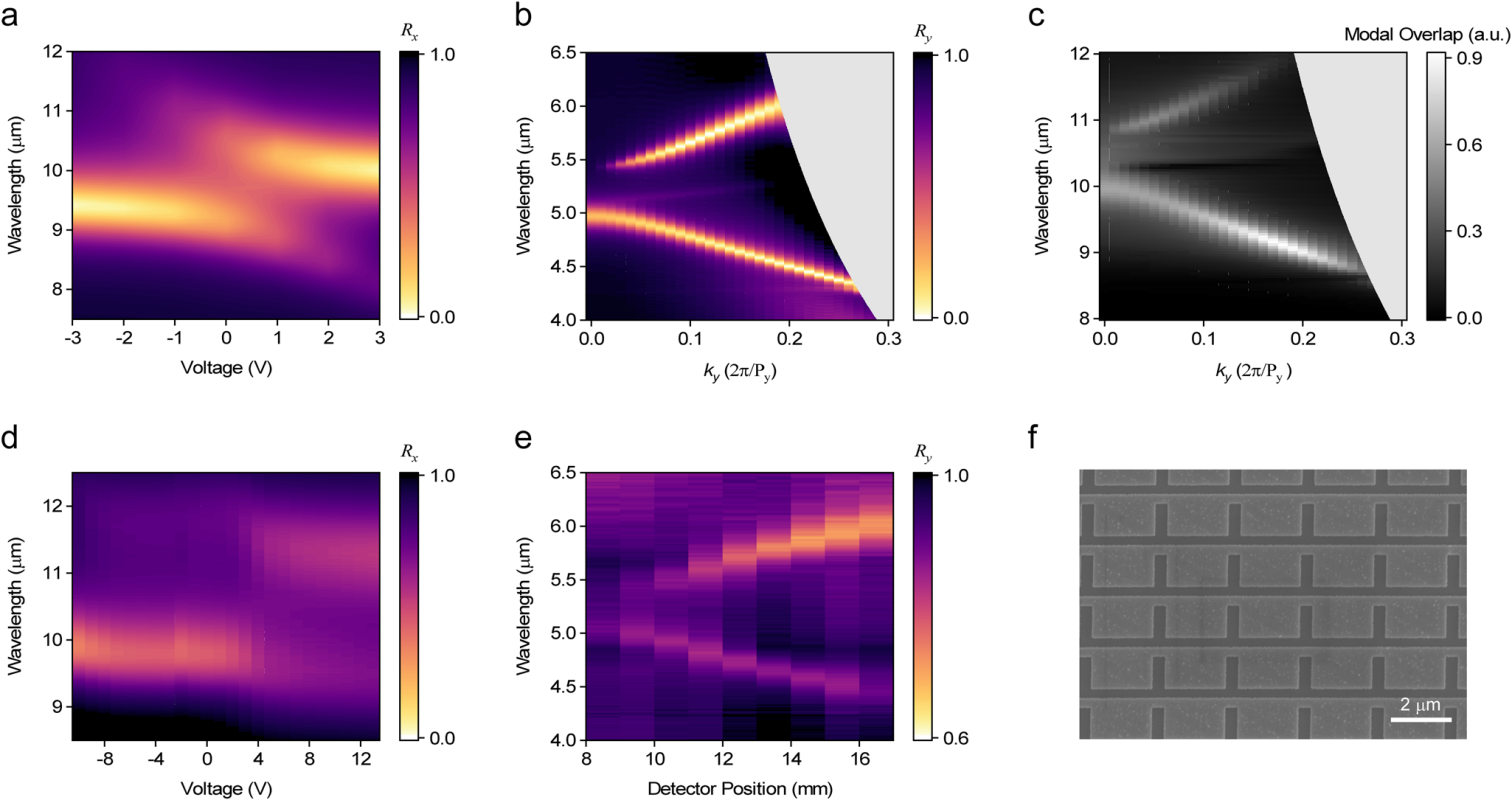
Linear characterization of dual tunability. a) Simulated x‐polarization reflection spectra of the local mode at the input pump wavelength as a function of applied voltage. b) Simulated y‐polarization reflection spectra of the nonlocal mode at the SH wavelength as a function of in‐plane momentum *k_y_*. The gray region represents the area below the light line. c) Calculated spectra of the modal overlap factor as a function of *k_y_*. d) Measured x‐polarization reflection spectra of the fabricated sample as a function of applied voltage. e) Measured y‐polarization reflection spectra of the fabricated sample as a function of detector position in the Fourier plane. f) SEM image of the fabricated sample.

To evaluate the dual tunability of the designed metasurface–namely, its response to both the incident angle and applied electric bias–we conducted simulations of the reflection spectra at both the FF and SH wavelengths as functions of the applied voltage and in‐plane momentum. When a vertical external electric field is applied to the MQW layer via the connected metal lines on the meta‐atoms, the IST frequency of the MQW shifts due to the QCSE. As a result, spectral tuning and polaritonic peak splitting are observed near the FF wavelength of 10 µm, arising from strong coupling between the IST and the LSPR, as shown in **Figure** **3**a. This demonstrates that both the absorption characteristics of the metasurface and the induced *E*_*z*_ field in the MQW layer are voltage‐dependent over a broad FF spectral range. Accordingly, in Equation (1), the term $E_{z}^{\omega }/E_{x,inc}^{\omega }$ can be considered as $E_{z}^{\omega }\left(V\right)/E_{x,inc}^{\omega }$. Moreover, since the IST frequency itself is voltage‐dependent, the nonlinear susceptibility can be expressed as $\chi_{zzz}^{(2)\mathrm{MQW}}\left(V\right)$, as previously reported in earlier studies.^[^ ^26^ ^]^ On the other hand, Figure 3b shows that at the SH wavelength of 5 µm, spectral tuning of the TM GMR mode occurs as *k_y_* (i.e. incident angle) is varied. The fact that both resonance branches shift with *k_y_* indicates that the field enhancement factor in Equation (1) can be expressed as $E_{z}^{2\omega }\left(2k_{y}\right)/E_{y,inc}^{2\omega }$, based on the in‐plane momentum matching condition *k*_*y*,SH_ =  2*k*_*y*,FF_. Previous studies have reported that the applied voltage has a negligible effect on the reflection spectrum in the SH region due to the weak IST between electron subbands 1 and 3.^[^ ^26^ ^]^ Additionally, it has been shown that the reflection spectrum of the FF LSPR mode is independent of in‐plane momentum under TE polarized light excitation.^[^ ^40^ ^]^ This mutual independence has been validated through our own simulations, and the corresponding results are provided in Figures S1 and S2 (Supporting Information). Consequently, FF and SH field components can be treated as nearly independent, and the modal overlap factor in Equation (1) can be expressed as a product of single‐variable functions. Figure 3c presents the modal overlap factor spectrum as a function of *k_y_* at 0 V. The non‐zero modal overlap, specifically engineered for the local‐to‐nonlocal SHG process, tracks the TM GMR branches in the SH region as *k_y_* varies. Simulation results illustrating how the modal overlap spectrum evolves with applied voltage are provided in Figure S3 (Supporting Information). As shown, the peak positions of the calculated modal overlap factors consistently follow the momentum dependence of the TM GMR branches under all voltage conditions. However, the magnitude of the overlap factor varies significantly with the applied voltage. This behavior indicates that electrical tuning primarily modulates the SH intensity, while angular tuning determines the SH spectral position, thereby confirming their independent tunability.

To experimentally verify the dual tunability, a 300 × 300 µm^2^ metasurface pattern was fabricated. Detailed fabrication procedures are described in Figure S4 (Supporting Information). Figure 3f presents a scanning electron microscopy (SEM) image of the fabricated sample. To characterize the electrical tuning behavior at the FF, reflection spectra were measured under x‐polarized incident light while sweeping the applied voltage from −10 to +13 V in 1 V increments. As shown in Figure 3d, polaritonic peak splitting was observed, in good agreement with the simulation results, although the measured spectral features are slightly redshifted compared to the simulations. To evaluate angular tuning at the SH wavelength, an angle‐resolved Fourier Transform Infrared (FTIR) measurement setup was constructed, wherein the detector position was translated across the Fourier plane in 1 mm steps to record the reflection spectra. Further details of the reflection‐type angle‐resolved FTIR setup are provided in Figure S6 (Supporting Information). As shown in Figure 3e, TM GMR branches appear under y‐polarized illumination, consistent with the simulation results. Compared to simulations, the nonlocal resonance dips are shallower and exhibit lower Q‐factors, likely due to the finite size of the fabricated sample and the incoherent nature of the thermal light source used in FTIR.^[^ ^41^ ^]^

### 2.3 Nonlinear Characterization of Dual Tunability

**Figure 4:**
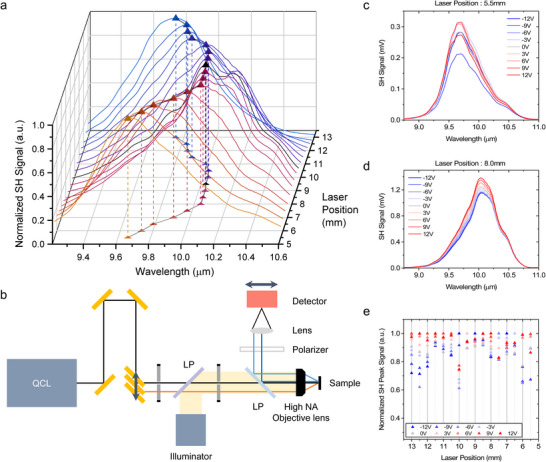
Nonlinear characterization of dual tunability. a) Measured SH signal spectra for various pump laser positions from 5.5 to 13.0 mm with 0.5 mm intervals. Tetrahedral markers represent the SH spectral peaks, and their projections illustrate the SH spectral tuning as a function of the incident laser position. The black marker corresponds to the SH spectral peak at the 9.5 mm of the pump laser position, representing normal incidence. b) Schematic of the reflection‐type angle‐resolved nonlinear measurement setup. c,d) Measured SH signal spectra at various bias voltages from −12 to 12 V for pump positions at c) 5.5 and d) 8.0 mm. e) SH peak intensity as a function of applied voltage for each pump laser position.

Unlike transmission‐type devices such as all‐dielectric metasurfaces, reflection‐type devices offer the advantage of efficient SH light collection through a single output port. However, they present challenges for angle‐resolved SH spectra measurements, as rotating the metasurface typically requires simultaneous rotation of the collimation and detection optics by 2θ for a sample rotation of θ. This necessitates a complex optical configuration and hinders continuous measurements due to potential interference between the objective and collimating lenses. Furthermore, unlike linear angle‐resolved measurements, nonlinear signals are inherently weak, rendering beam‐spreading techniques such as Fourier imaging unsuitable. To address these challenges, we implemented a reflection‐type angle‐resolved nonlinear measurement setup, as illustrated in **Figure** **4**b. Instead of rotating the sample, the incident angle of the pump beam was varied by laterally displacing the beam using a mirror mounted on a translation stage in combination with a high numerical aperture (NA) aspherical objective lens. Figure 4a shows the measured SH spectral tuning as a function of the pump beam position, which corresponds to the incident angle. The laser position corresponding to normal incidence (θ  =  0°) was determined to be 9.5 mm. From the projected trajectory of the SH spectral peak in the xy‐plane, we observe that the SH peak wavelength shifts from 10.04 to 9.66 µm as |*k_y_* | increases. To confirm that the observed SH angular dependence originates from the nonlocal mode, we compared the measured SH spectrum with the angle‐dependent reflection spectrum in the SH region. At normal incidence (laser position = 9.5 mm), the SH peak wavelength was 5.02 µm, while at the maximum incident angle (laser position = 5.5 mm), it shifted to 4.83 µm. Referring to Figure 3b, we observe that the GMR dip occurs at 4.83 µm for a momentum of *k_y_* =  0.1. To relate the laser position shift to the incident angle, we calculate $\theta =\tan^{-1}\frac{9.5\;\mathrm{mm}-5.5\;\mathrm{mm}}{f}=17.48^{\circ }$, where *f*  =  12.7 mm is the focal length of the aspherical lens. The corresponding normalized in‐plane momentum is given by $k_{y}=\frac{P_{y}}{\lambda_{\mathrm{SH}}}\;\sin\theta =0.106$, which is in excellent agreement with the linear simulation results. Moreover, the nearly constant SH peak wavelength near *k_y_* =  0 matches the behavior observed in Figure 3b, further confirming that the SH spectral tuning arises from the nonlocal resonance at the SH frequency. Due to the size of the optics used in our current experimental setup, the maximum achievable incident angle was restricted to 17.48°. However, broader angular and spectral tunability could be achieved using optics with larger diameter (see also Figure S9, Supporting Information).

Figure 4c,d show the measured SH spectral tuning at two different laser positions, with the applied voltage varied from −12 to +12 V in 3 V increments. Notably, in both cases, the SH peak wavelength remained nearly constant, while only the peak intensity was modulated and the overall spectral shape was preserved (see also Figure S7, Supporting Information). This behavior can be attributed to the low Q‐factor of the LSPR and the broad spectral tunability of the polaritonic resonance resulting from strong coupling with the IST, as observed in Figure 3d. The LSPR spectrum undergoes a broad shift from 9 to 12.5 µm with varying applied voltage. Because the nonlocal SH mode exhibits a high Q‐factor determined by the laser position, it acts as a narrow spectral filter that selectively probes voltage‐induced variations in pump absorption and field enhancement at the FF. Consequently, voltage‐induced modulation of the SH intensity originates from the local FF mode. Figure 4e presents the SH peak intensity as a function of applied voltage for each laser position, clearly demonstrating that the local‐to‐nonlocal SHG process enables independent control of the SH peak wavelength (via angle) and SH intensity (via voltage). The MQW layer used in this work was originally designed to minimize detuning between the 1–2 and 2–3 ISTs under applied bias.^[^ ^39^ ^]^ As a result, the peak value of the nonlinear susceptibility $|\chi_{zzz}^{\left(2\right)}\left(V\right)|$ remains relatively stable across the voltage range, limiting the achievable modulation depth to ≈36% in the fabricated device. As shown in previous work, if the MQW structure is redesigned with the same total thickness but optimized to exhibit stronger voltage‐dependent modulation of $|\chi_{zzz}^{(2)\mathrm{MQW}}\left(V\right)|$ by detuning transition energies (see also Figure S8, Supporting Information), it is possible to realize significantly high SH intensity modulation.

## 3 Conclusion

In this work, we demonstrated a local‐to‐nonlocal SHG scheme that enables independent control of SH spectral position and intensity. A central outcome of this approach is the exploitation of nonlocal metasurfaces for angle‐dependent SHG spectral tuning, a capability that has remained largely unexplored despite extensive studies on their high‐Q resonances. By combining a local plasmonic resonance at the fundamental frequency with a nonlocal guided‐mode resonance at the SH frequency, our design decouples the excitation and emission pathways. This dual‐mode strategy enhances flexibility in nonlinear metasurface design, allowing spectral tuning and intensity modulation to be independently addressed. Importantly, unlike local‐only or nonlocal‐only approaches used in previous studies for nonlinear metasurfaces, this local‐to‐nonlocal scheme can mitigate the trade‐offs that typically arise between tunability and efficiency.

While intersubband polaritonic metasurfaces are renowned for their exceptionally strong nonlinear responses, their intrinsic optical losses have hindered their integration into nonlocal architectures. Our local‐to‐nonlocal SHG strategy overcomes this challenge: it preserves the strong nonlinearity and electrical tunability of polaritonic systems, while concurrently leveraging the angle‐dependent spectral control inherent to nonlocal metasurfaces. This combination provides a general framework for realizing nonlinear metasurfaces with enhanced versatility, offering new opportunities for nonlinear signal processing, angle‐multiplexed photonics, and quantum light generation.

## 4 Experimental Section

### Numerical Simulation

The meta‐atom simulations were performed using a finite‐difference time‐domain (FDTD) solver (Lumerical FDTD). The structure shown in Figure 2a was modeled with Bloch boundary conditions applied in the x and y directions, and a perfectly matched layer (PML) in the z direction. *k_y_* of the Bloch boundary was swept from 0 to 0.3 to simulate incident angle dependence for both local and nonlocal modes. The material information of the MQW layer was obtained from the previous study.^[^ ^39^ ^]^

### Device Fabrication

A 500‐nm‐thick MQW layer, epitaxially grown on an InP substrate, was transferred onto a Si substrate via thermo‐compression wafer bonding. Prior to bonding, 20 nm Cr/50 nm Pt/150 nm Au were sequentially deposited by electron‐beam evaporation onto both the MQW and Si wafers to form the bonding interface and the bottom metal plane. The InP substrate was removed through mechanical polishing and selective chemical etching, using etch‐stop layers consisting of 300 nm In_0.53_Ga_0.47_As and 100 nm InP. The top metal nanoantennas, composed of 6 nm Cr and 60 nm Au, were patterned by electron‐beam lithography followed by lift‐off process after metal deposition via electron‐beam evaporation. For active device structuring, 400  ×  400 µm^2^ mesa patterns were defined by photolithography and etched by inductively coupled plasma (ICP) etching, using a 460 nm Si_x_N_y_ mask layer to reduce current leakage. A 330 nm Si_x_N_y_ passivation layer was deposited, and top patterned regions were opened by photolithography and a buffered oxide etchant. Top contact electrodes composed of 20 nm Cr/300 nm Au/20 nm Cr/50 nm Au were deposited and patterned by lift‐off process after photolithography. The fabricated device was mounted on a copper heat sink using silver paste. A detailed illustration of the fabrication process is provided in the Supporting Information.

### Optical Characterization

Voltage‐dependent reflection spectra under x‐polarized illumination were measured using a FTIR spectrometer equipped with an infrared microscope (Bruker VERTEX70 and HYPERION 1000) and a source meter (Keithley SMU 2450). Angle‐resolved reflection spectra under y‐polarized illumination were obtained using an external Fourier plane imaging setup integrated with the FTIR system, as detailed in the Supporting Information. Thermal light was directed through a polarizer, a CaF_2_ beam splitter, and a high‐NA ZnSe aspherical objective lens (NA: 0.67, focal length: 12.7 mm). The reflected light was separated at the beam splitter, passed through ZnSe lenses (focal length: 75 mm), and collected by an MCT detector (InfraRed Associates, Inc.) placed behind a 0.5 mm pinhole at the Fourier plane. For nonlinear optical measurements, a pulsed QCL (Daylight Solutions, tuning range: 909–1230 cm^−1^, peak power: 150 mW, repetition rate: 100 kHz, duty cycle: 10%) was used with an InSb photodetector (Electro‐Optical Systems, Inc.). The linearly polarized QCL beam was directed onto the sample after reflected by a mirror mounted on a motorized moving stage, passed through a LP filter (cutoff wavelength: 7 µm) before being focused onto the metasurface by the ZnSe objective lens. The generated SH signal was collected by the detector after reflected from the LP filter, passing through a polarizer, and a ZnSe lens (focal length: 25.4 mm). The focal spot diameter at the sample was measured to be 2w = 48 µm using the knife‐edge method. A DC bias was applied using the source meter while scanning the laser position.

## Conflict of Interest

The authors declare no conflict of interest.

## Supporting information



Supporting Information

## Data Availability

The data that support the findings of this study are available from the corresponding author upon reasonable request.
