# Supplementary material for: Local‐to‐Nonlocal Second‐Harmonic Generation from Electrically Tunable Intersubband Polaritonic Metasurfaces
Source: Adv Sci (Weinh). 2025 Nov 29;13(9):e18776. doi: 10.1002/advs.202518776 (PMC12904043; doi:10.1002/advs.202518776)
Supplement: Supplementary file 1 — Supporting Information [file ADVS-13-e18776-s001.docx]

Supporting Information

Local-to-Nonlocal Second-Harmonic Generation from Electrically Tunable Intersubband Polaritonic Metasurfaces

Jaesung Kim, Hyeongju Chung, Seongjin Lee, Gerhard Boehm, Mikhail A. Belkin, and Jongwon Lee^*^

Figures S1 - S11


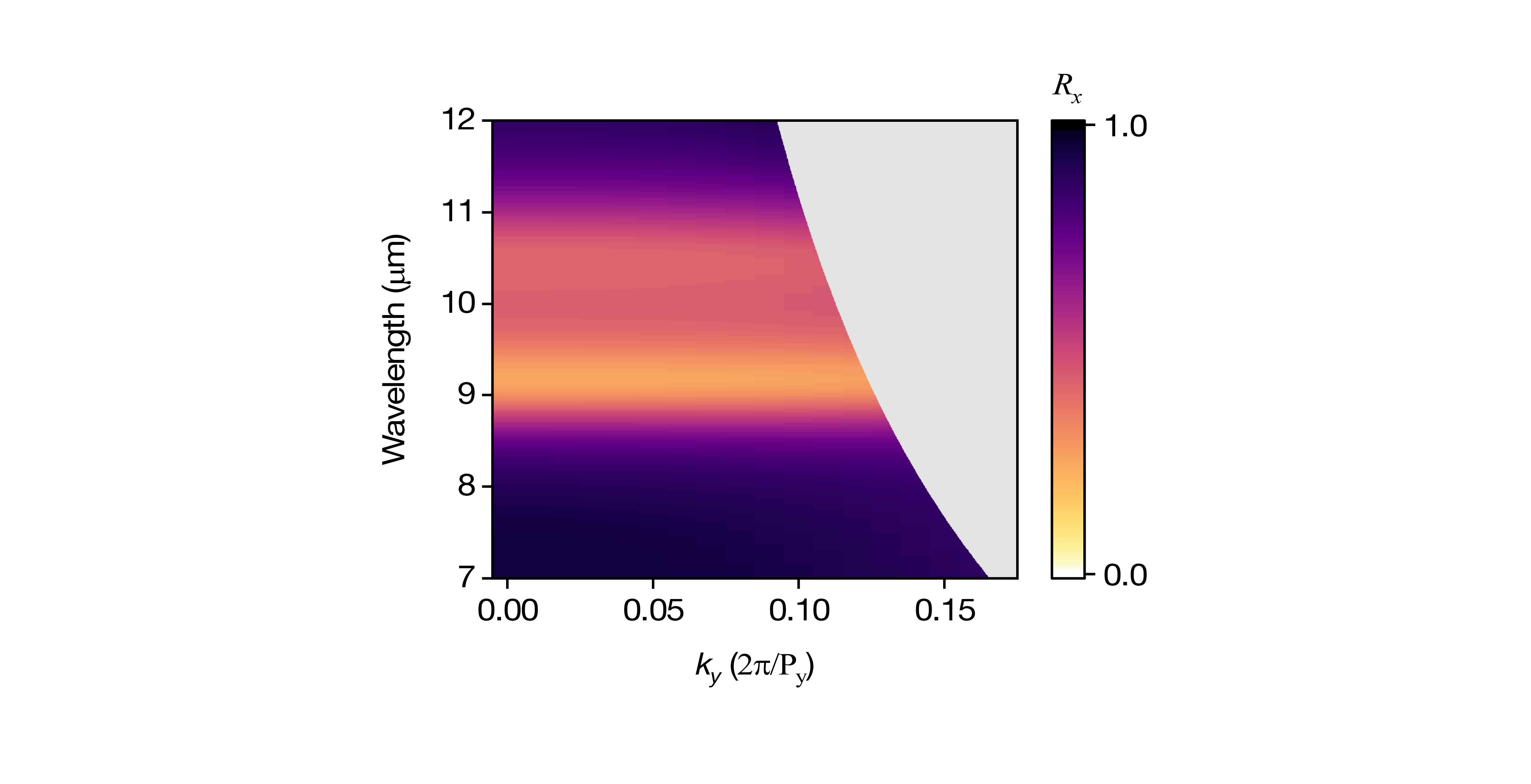


**Figure S1.** Angle-independence of the local mode at the FF wavelength.


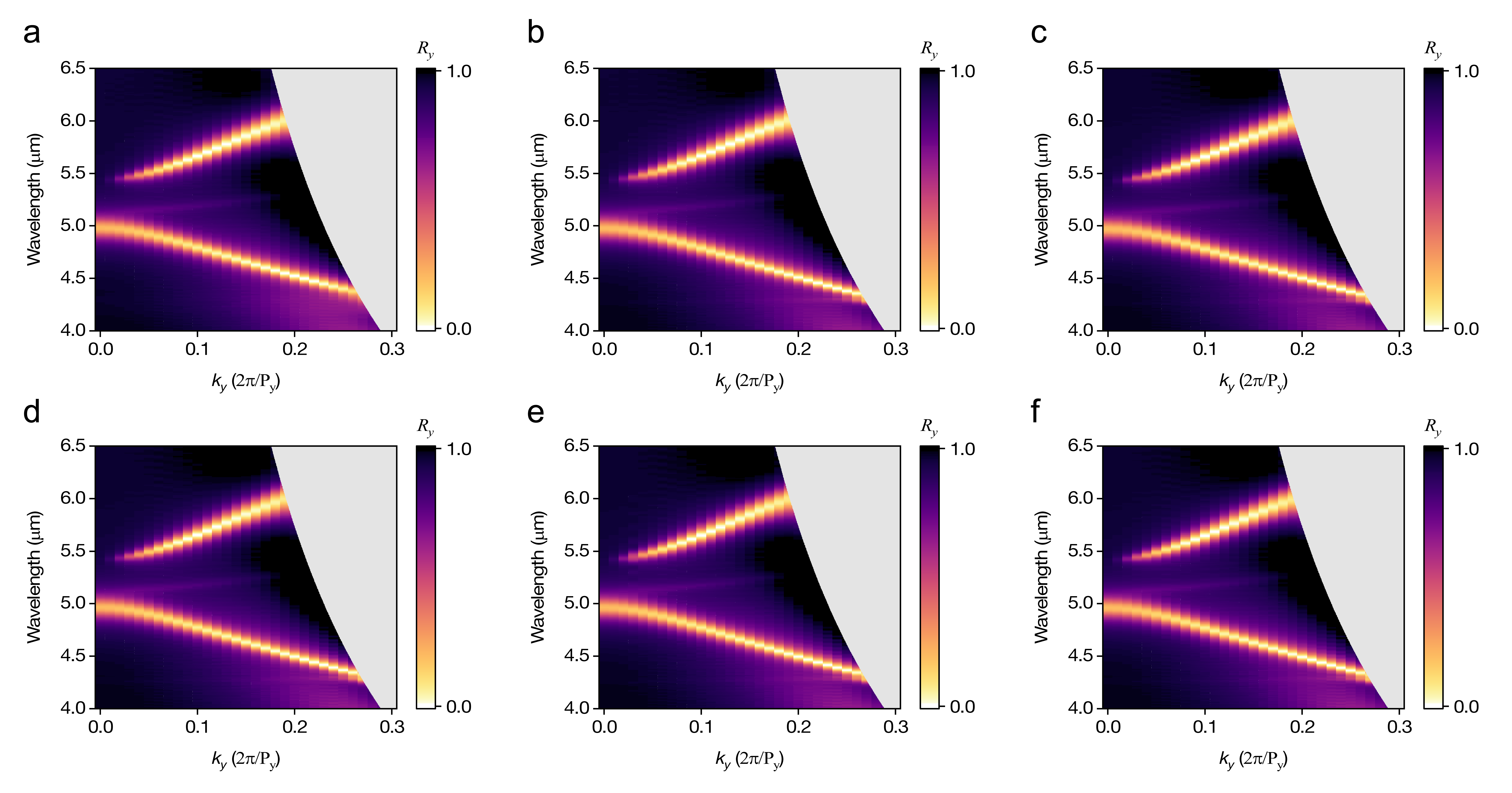


**Figure S2.** Voltage-independence of the nonlocal mode at the SH wavelength. Each panel corresponds to **a)** -3 V, **b)** -2 V, **c)** -1 V, **d)** 1 V, **e)** 2 V, and **f)** 3 V.


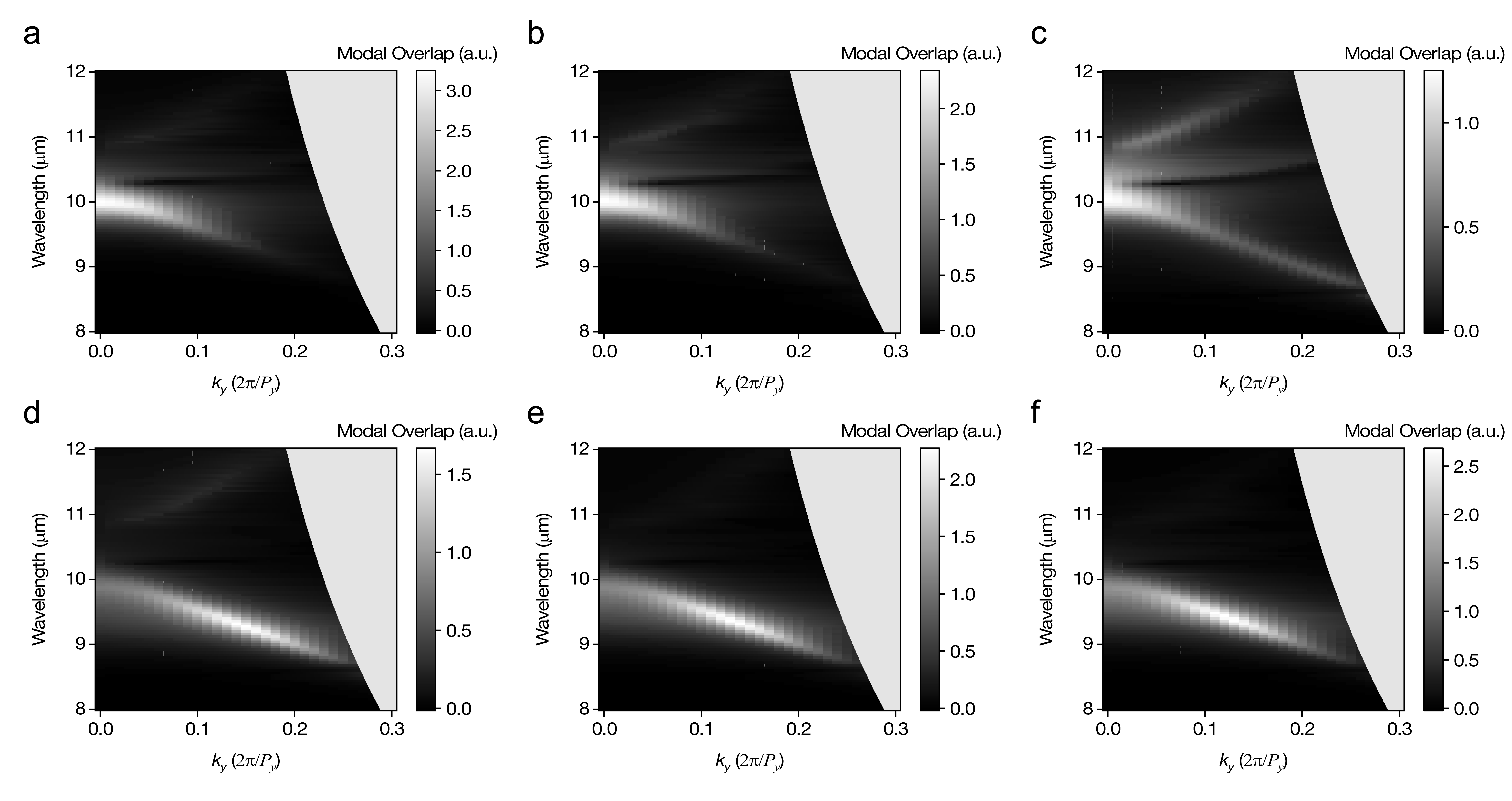


**Figure S3.** Angle and voltage dependence of the calculated modal overlap factor. Each panel corresponds to **a)** -3 V, **b)** -2 V, **c)** -1 V, **d)** 1 V, **e)** 2 V, and **f)** 3 V.


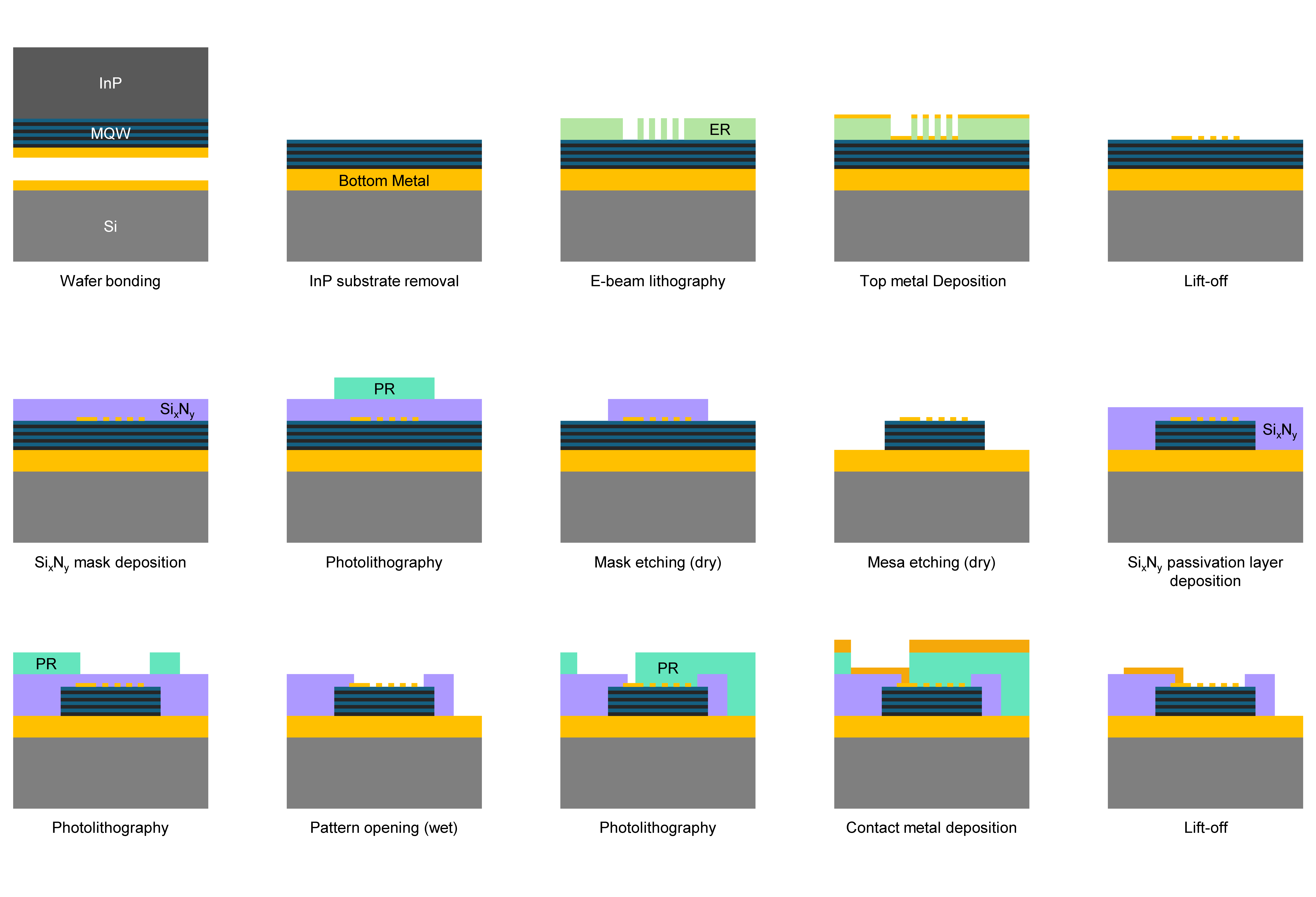


**Figure S4.** Schematic of the metasurface fabrication process.


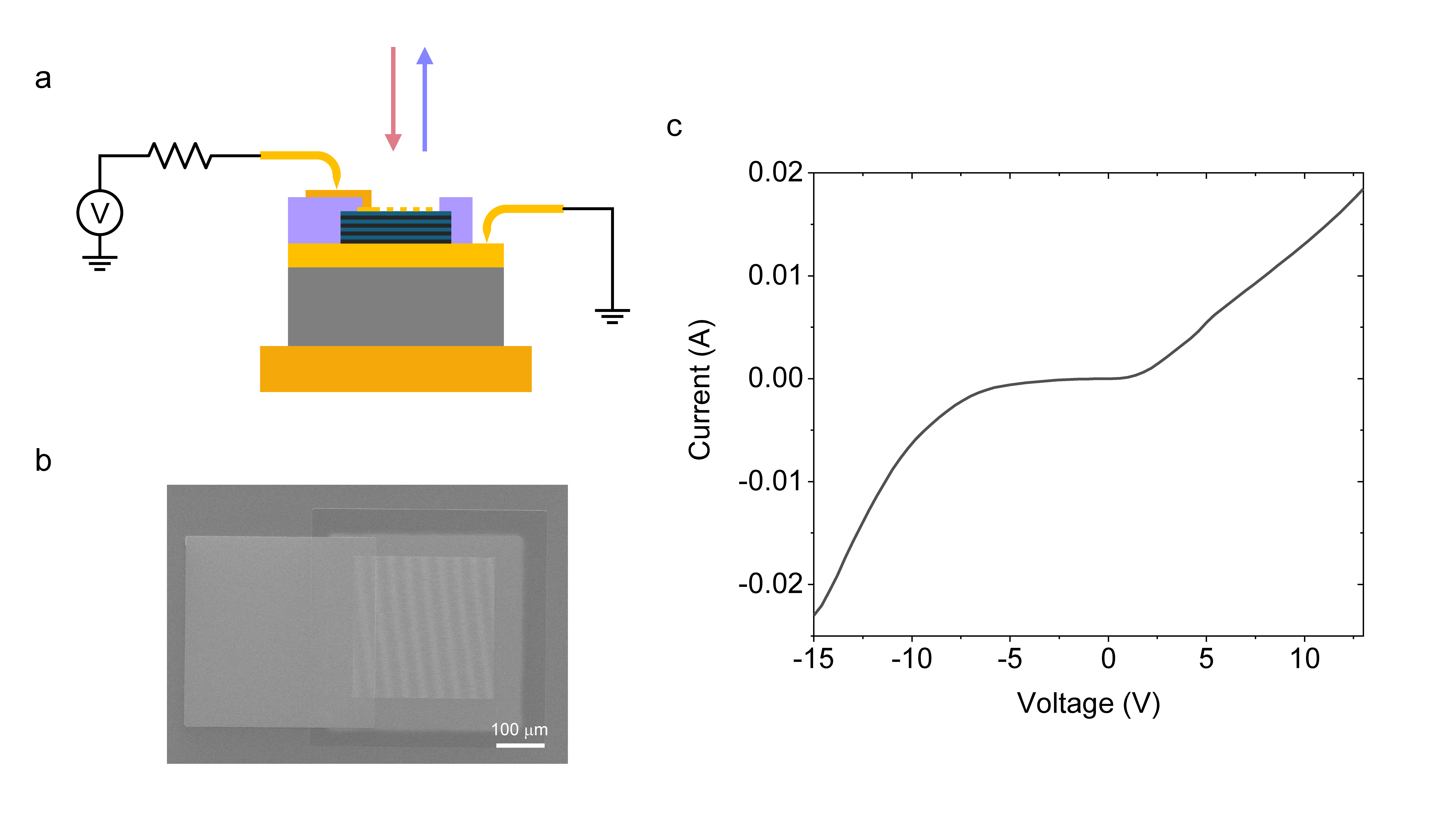


**Figure S5.** I-V characterization. **a)** Illustration of the voltage tuning measurement setup. **b)** SEM image of the fabricated sample for voltage tuning. **c)** Measured I-V characteristics of the fabricated sample.


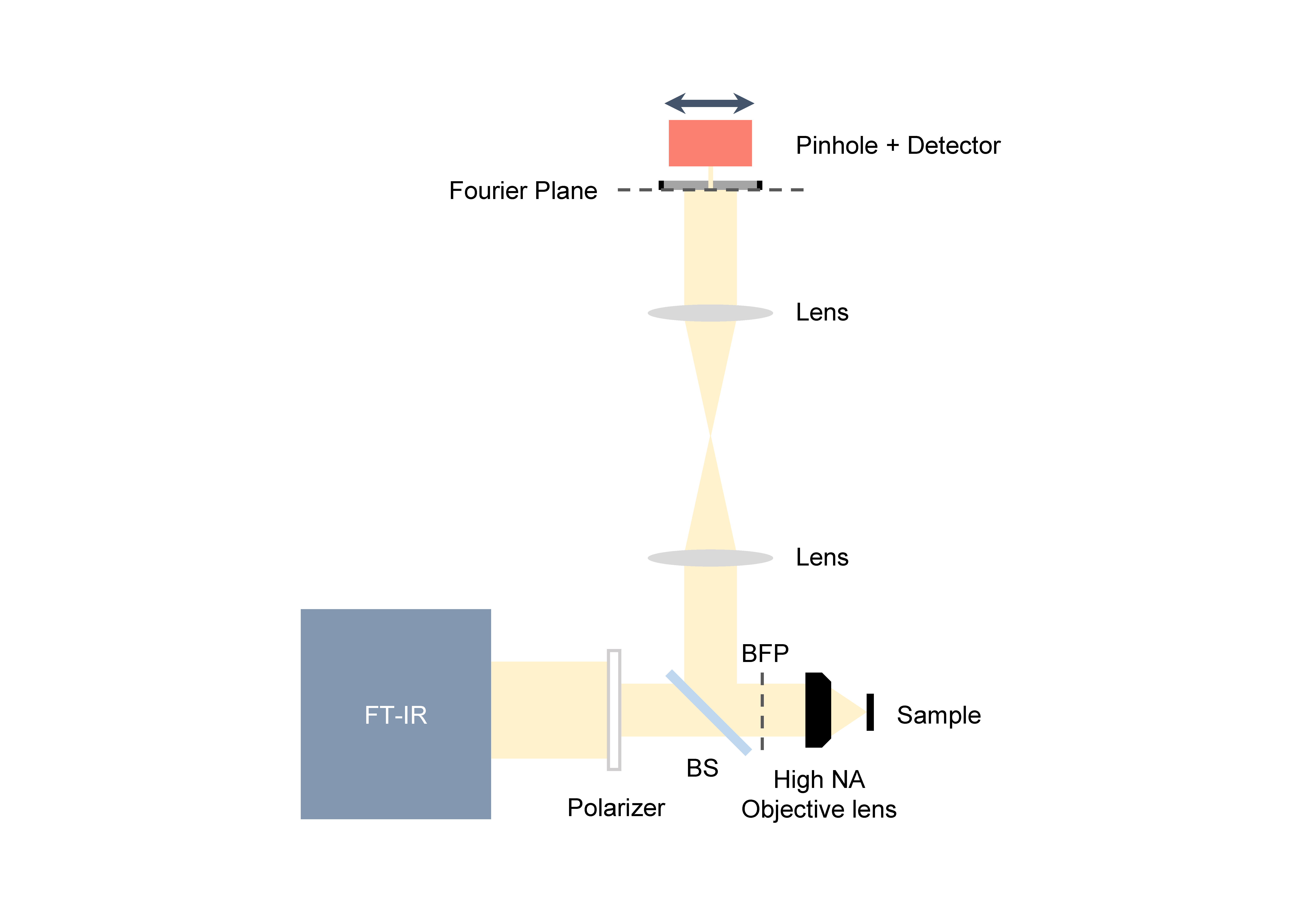


**Figure S6.** Reflection-type angle-resolved FT-IR measurement setup.





**Figure S7.** Comparison of the impact of dual tunability enabled by local-to-nonlocal SHG process. The panels on the left side represent normalized SH spectra, and the panels on the right side represent accessible peak positions of normalized SH spectra. **top)** Voltage tuning of SH spectra (single tunability). **middle)** Angle tuning of SH spectra (single tunability). **bottom)** Angle, voltage tuning of SH spectra (dual tunability).


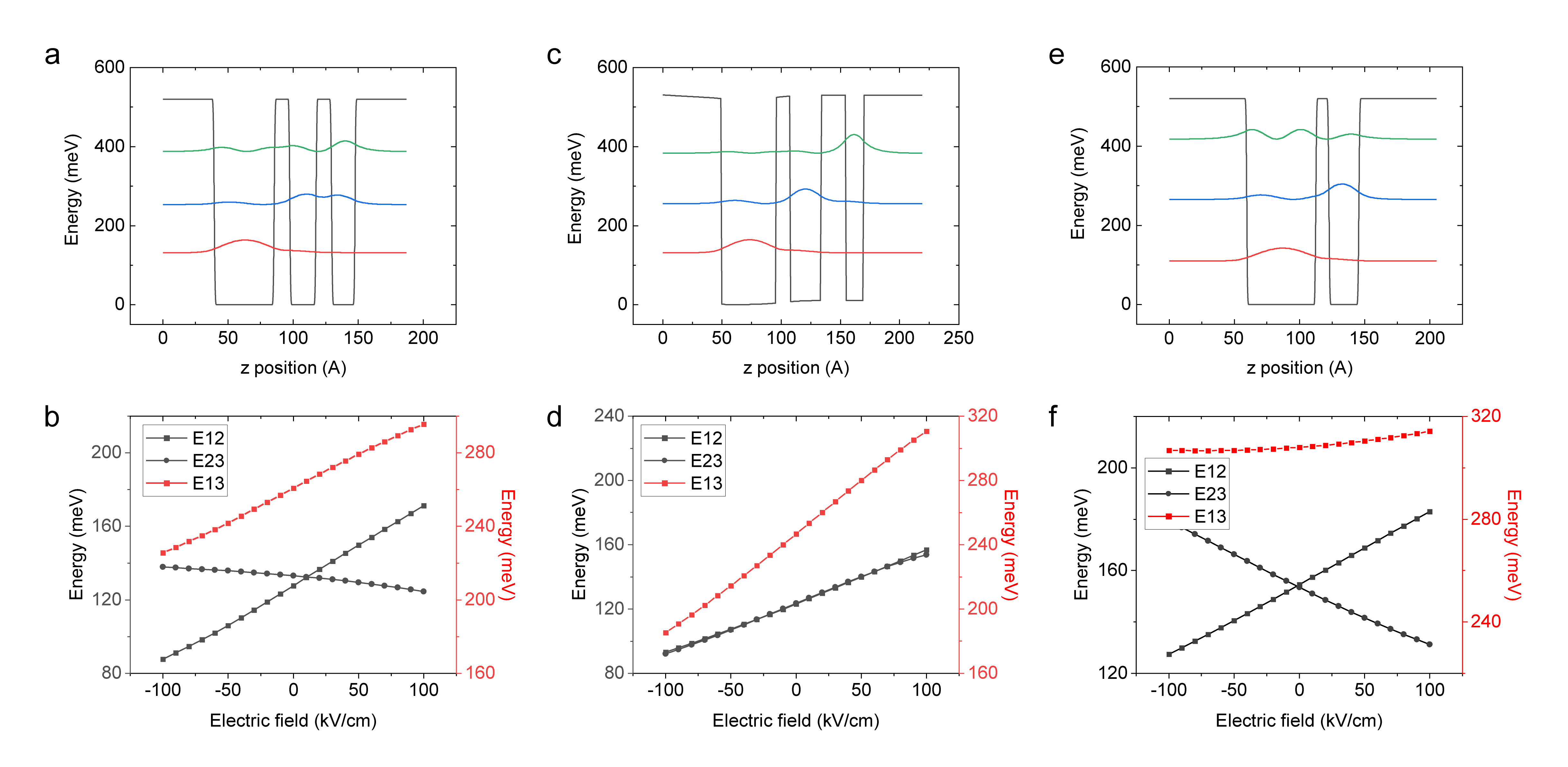
**Figure S8.** Comparison of the MQW designs and corresponding energy level detuning under electric bias. Each MQW design is studied in **a,b)** [Ref S1] **c,d)** [Ref S2] **e,f)** [Ref S3].


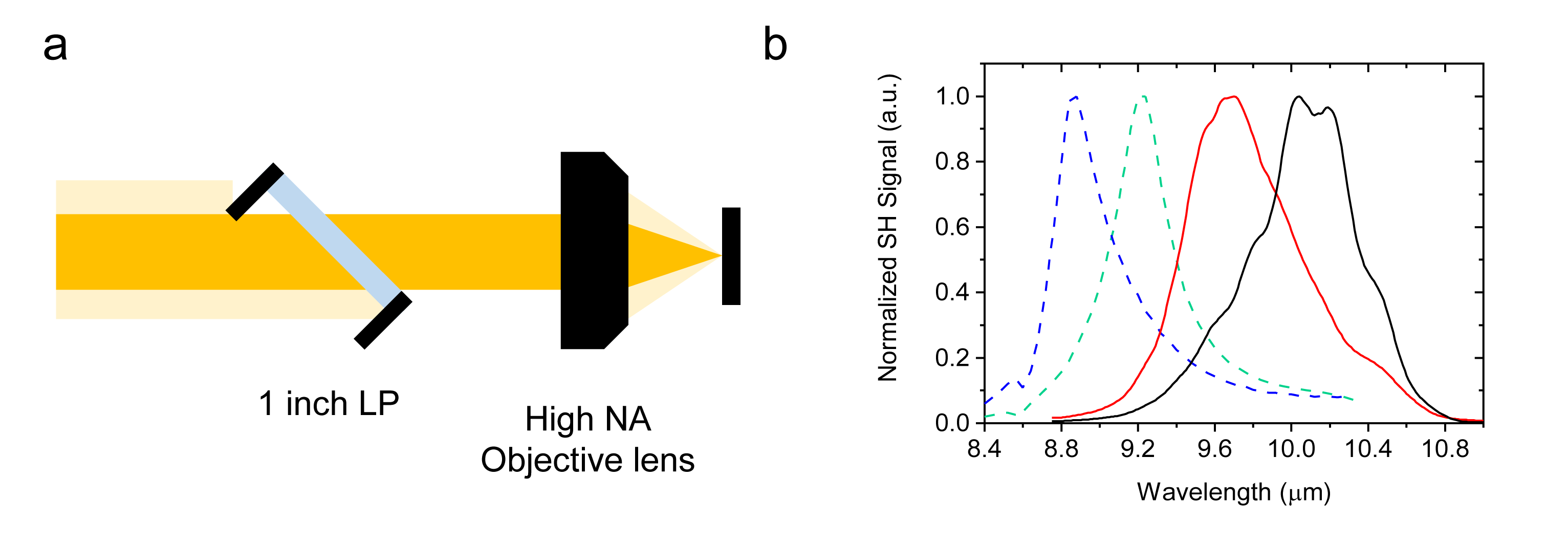


**Figure S9.** **a)** Illustration of the maximum angle limitation due to optical devices. **b)** Measured SH spectra (solid line) at 0° (black) and 17.48° (red) with simulated SH spectra (dashed line) at 25.77° (green) and 36.92° (blue).


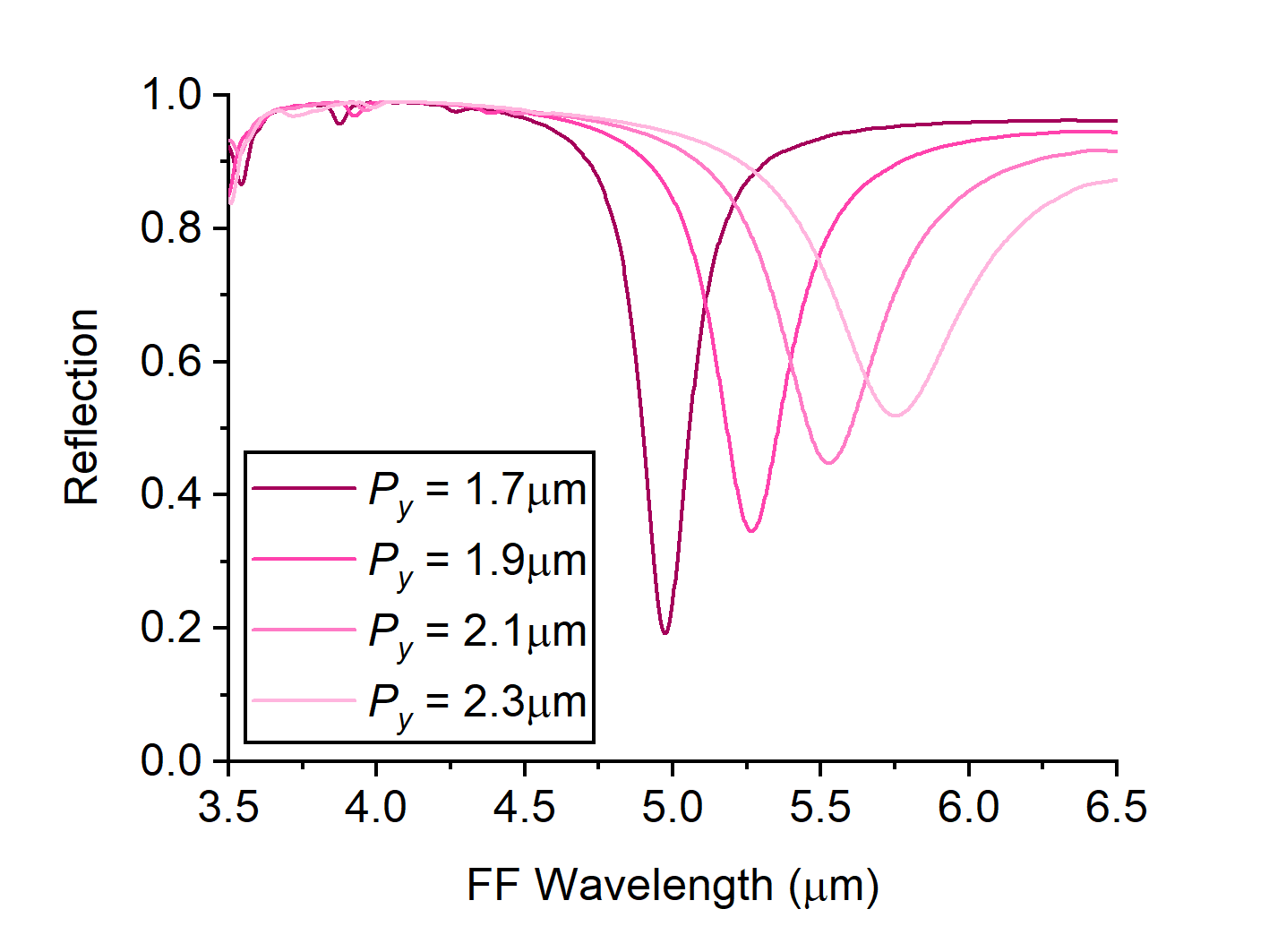


**Figure S10.** Q-factor degradation of TM-GMR by varying $P_{y}$ at fixed $t_{\mathrm{MQW}}$.


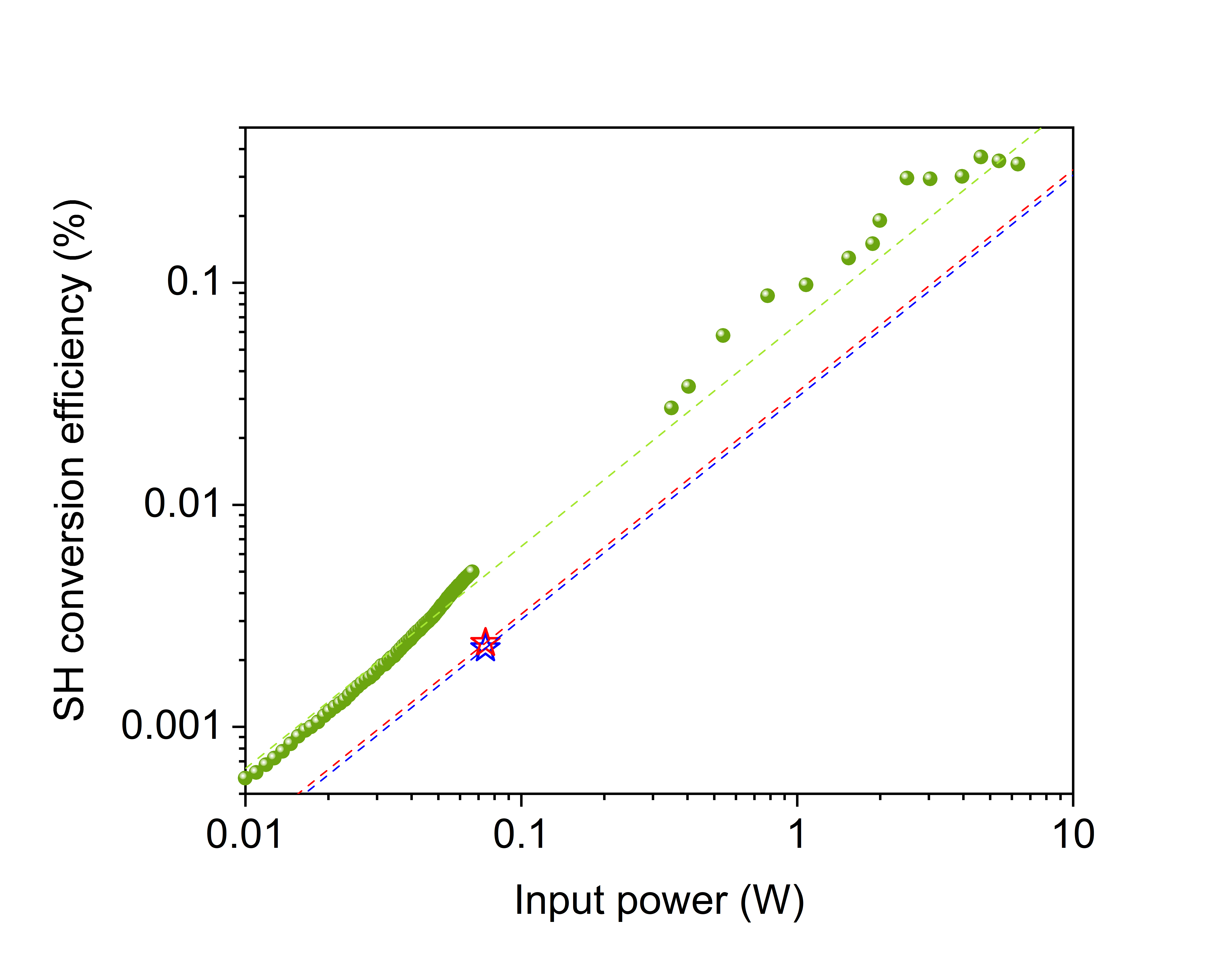


**Figure S11.** Comparison of SH conversion efficiency. The green dots are the data from the previous study which reported record-high SH-conversion efficiency [Ref S4]. The red star is the data from this work. The blue star is the data from the previous study which used same MQW design [Ref S2]. The dashed lines represent trend of SH conversion efficiency proportional to input pump power.

**References**

[S1] Yu, J., S. Park, I. Hwang, G. Boehm, M. A. Belkin, and J. Lee. "Broadband Giant Nonlinear Response Using Electrically Tunable Polaritonic Metasurfaces." 13, no. 7 (2024): 1131–39. https://doi.org/doi:10.1515/nanoph-2023-0682.

[S2] Yu, J., J. Kim, H. Chung, et al. "Full Complex Amplitude Control of Second-Harmonic Generation Via Electrically Tunable Intersubband Polaritonic Metasurfaces." *Science Advances* 11, no. 30: eadw8852. https://doi.org/10.1126/sciadv.adw8852.

[S3] Lee, J., M. Tymchenko, C. Argyropoulos, et al. "Giant Nonlinear Response from Plasmonic Metasurfaces Coupled to Intersubband Transitions." *Nature* 511, no. 7507 (2014): 65–69. https://doi.org/10.1038/nature13455.

[S4] Kim, D., J. Yu, G. Boehm, M. A. Belkin, and J. Lee. "Efficient Second-Harmonic Generation from Dielectric Inter-Subband Polaritonic Metasurfaces Coupled to Lattice Resonance." *Nano Letters* 23, no. 19 (2023): 9003–10. https://doi.org/10.1021/acs.nanolett.3c02626.
